# Supplementary material for: The Relative Abundance and Occurrence of Sharks off Ocean Beaches of New South Wales, Australia
Source: Biology (Basel). 2022 Oct 4;11(10):1456. doi: 10.3390/biology11101456 (PMC9599013; doi:10.3390/biology11101456)

Supporting Information

Table S1: The names and locations of study beaches in New South Wales, Australia, and the surveying periods for drone flights conducted between 2017 – 2021.

Location	Beach name	Location co-ordinates	Survey periods
Kingscliff	Kingscliff	-28.255125, 153.578903	4 Nov 2018 – 25 Apr 2021
Kingscliff	South Kingscliff	-28.273453, 153.581517	24 Sep 2019 – 30 Dec 2020
Cabarita	Cabarita	-28.331628, 153.572042	13 Sep 2020 – 25 Apr 2021
Byron Bay	Main	-28.640769, 153.614531	28 Dec 2017 – 15 Apr 2021
Byron Bay	The Pass	-28.636925, 153.627922	3 Feb 2018 – 28 Apr 2019
Suffolk Park	Suffolk Park	-28.690917, 153.614656	3 Feb 2018 – 26 Apr 2019
Lennox Head	Seven Mile	-28.785224, 153.594193	3 Feb 2018 – 25 Apr 2021
Ballina	Sharpes	-28.833919, 153.605117	4 Feb 2018 – 25 Apr 2021
Ballina	Shelly	-28.864428, 153.593856	4 Feb 2018 – 24 Apr 2021
Ballina	Lighthouse	-28.868550, 153.592258	10 Feb 2018 – 23 Apr 2021
Evans Head	Airforce	-29.102372, 153.433156	3 Apr 2021 – 25 Apr 2021
Evans Head	Main	-29.111994, 153.434939	3 Feb 2018 – 27 Jan 2021
Yamba	Main	-29.435178, 153.364764	18 Nov 2018 – 25 Apr 2021
Woolgoolga	Woolgoolga	-30.109186, 153.203319	20 Dec 2018 – 28 Apr 2019
Sawtell	Sawtell	-30.368325, 153.103200	28 Sep 2020 – 25 Apr 2021
Urunga	Hungry Head	-30.522644, 153.028556	12 Dec 2020 – 27 Jan 2021
Scotts Head	Scotts Head	-30.744669, 152.996953	19 Dec 2020 – 27 Jan 2021
South West Rocks	South West Rocks	-30.883297, 153.043347	20 Dec 2020 – 5 Feb 2021
Crescent Head	Killick	-31.186519, 152.978986	17 Nov 2019 – 25 Apr 2021
Port Macquarie	Town	-31.429797, 152.920536	12 Mar 2019 – 18 Mar 2019

Port Macquarie	Flynns	-31.442536, 152.926233	8 Dec 2018 – 26 Apr 2020
Port Macquarie	Lighthouse	-31.476125, 152.933597	30 Dec 2018 – 25 Apr 2021
Forster	Main	-32.177531, 152.512414	23 Nov 2019 – 25 Apr 2021
Hawks Nest	Bennetts	-32.673558, 152.186017	13 Dec 2020 – 26 Jan 2021
Anna Bay	Birubi	-32.784086, 152.075364	21 Dec 2018 – 25 Apr 2021
Newcastle	Nobbys	-32.924617, 151.792944	27 Sep 2020 – 23 Apr 2021
Lake Macquarie	Redhead	-33.014144, 151.720436	4 Nov 2018 – 25 Apr 2021
Lake Macquarie	Blacksmiths	-33.080125, 151.658050	22 Nov 2018 & 13 Dec 2020
North Entrance	North Entrance	-33.334436, 151.507025	26 Sep 2020 – 25 Apr 2021
Avoca	Avoca	-33.470342, 151.436928	9 Dec 2018 – 25 Apr 2021
Palm Beach	Palm Beach	-33.598889, 151.325447	26 Sep 2020 – 25 Apr 2021
South Narrabeen	South Narrabeen	-33.722917, 151.299572	3 Oct 2020 – 25 Apr 2021
Dee Why	Dee Why	-33.752422, 151.297314	26 Sep 2020 – 24 Apr 2021
Maroubra	Maroubra	-33.947550, 151.257750	28 Sep 2020 – 25 Apr 2021
Cronulla	Wanda	-34.043639, 151.161967	27 Sep 2020 – 25 Apr 2021
Coledale	Coledale	-34.288353, 150.948872	20 Dec 2020 – 26 Jan 2021
Shellharbour	Shellharbour North	-34.571972, 150.868444	21 Dec 2020 – 26 Jan 2021
Kiama	Surf	-34.676197, 150.854728	1 Dec 2018 – 26 Jan 2021
Mollymook	Mollymook	-35.338836, 150.474503	8 Dec 2018 – 26 Jan 2021
Malua Bay	Malua Bay	-35.793861, 150.230339	21 Dec 2019 – 27 Jan 2021
Tathra	Tathra	-36.726817, 149.982908	20 Dec 2018 – 27 Jan 2021
Pambula	Pambula	-36.941239, 149.910122	21 Dec 2018 – 27 Jan 2021

Table S2: Study beach locations and the number of drone flight days per sampling season.
*Please note: The beach name is only specified if it differs from the location name.

Location + (Beach name)	Season 1 2017/2018	Season 2 2018/2019	Season 3 2019/2020	Season 4 2020/2021
Kingscliff	0	82	2	72
Kingscliff (South)	0	0	99	6
Cabarita	0	0	0	98
Byron Bay (The Pass)	9	78	0	0
Byron Bay (Main)	7	84	120	42
Suffolk Park	6	68	0	0
Lennox Head (Seven Mile)	8	83	102	90
Ballina (Sharpes)	8	81	82	61
Ballina (Shelly)	9	61	91	75
Ballina (Lighthouse)	7	62	100	71
Evans Head (Airforce)	0	0	0	12
Evans Head (Main)	6	64	113	61
Yamba (Main)	0	42	85	96
Woolgoolga	0	53	0	0
Sawtell	0	0	0	60
Urunga (Hungry Head)	0	0	0	33
Scotts Head	0	0	0	38
South West Rocks	0	0	0	32
Crescent Head (Killick)	0	0	50	68
Port Macquarie (Town)	0	2	0	0
Port Macquarie (Flynns)	0	51	36	0
Port Macquarie (Lighthouse)	0	1	0	59
Forster (Main)	0	0	76	97
Hawks Nest (Bennetts)	0	0	0	28
Anna Bay (Birubi)	0	36	74	50
Newcastle (Nobbys)	0	0	0	64
Lake Macquarie (Redhead)	0	32	72	66
Lake Macquarie (Blacksmiths)	0	1	0	1
North Entrance	0	0	0	76
Avoca	0	59	0	102
Palm Beach	0	0	0	78
South Narrabeen	0	0	0	62
Dee Why	0	0	0	63
Maroubra	0	0	0	65
Cronulla (Wanda)	0	0	0	56
Coledale	0	0	0	32
Shellharbour (North)	0	0	0	30
Kiama (Surf)	0	32	19	30
Mollymook	0	25	14	35
Malua Bay	0	0	15	34
Tathra	0	18	22	38
Pambula	0	29	14	27
**Total**	**60**	**1044**	**1186**	**2008**

Table S3: Shark observation days, flights days and sightings per day at each beach location. Observation days include all days that a sighting occurred, and a flight day equals 24 hours of continuous flying time. Sightings per unit effort is measured as the ratio of observation days to flight days. Please note: The name of the study beach is only specified if it differs from the location name.

Location + (Beach name)	Observation days	Flight days	Sightings per unit effort
Forster (Main)	25	17.5	1.4
Pambula	4	4.8	0.8
Hawks Nest (Bennetts)	2	2.5	0.8
Crescent Head (Killick)	10	12.6	0.8
Ballina (Sharpes)	12	18.2	0.7
Scotts Head	3	5.1	0.6
Anna Bay (Birubi)	10	18.2	0.6
Lennox Head (Seven Mile)	13	23.8	0.5
Ballina (Lighthouse)	8	20.6	0.4
Port Macquarie (Lighthouse)	2	5.6	0.4
Cabarita	4	11.8	0.3
Kingscliff	5	15.3	0.3
Tathra	2	6.3	0.3
Kingscliff (South)	3	9.5	0.3
Newcastle (Nobbys)	2	7.1	0.3
Mollymook	2	7.2	0.3
Ballina (Shelly)	6	22.1	0.3
Sawtell	1	4.9	0.2
Byron Bay (Main)	3	19.9	0.2
Kiama (Surf)	1	8.4	0.1
Port Macquarie (Flynns)	1	8.9	0.1
Evans Head (Main)	2	23.0	0.1
Avoca	1	24.3	0.0
Yamba (Main)	0	22.5	0.0
Lake Macquarie (Redhead)	0	17.0	0.0
Palm Beach	0	11.1	0.0
Entrance North	0	10.6	0.0
South Narrabeen	0	6.8	0.0
Maroubra	0	6.6	0.0
Dee Why	0	6.5	0.0
Cronulla (Wanda)	0	5.9	0.0
Urunga (Hungry Head)	0	5.0	0.0
Byron Bay (The Pass)	0	4.5	0.0
Malua Bay	0	4.4	0.0
Suffolk Park	0	4.2	0.0
Coledale	0	4.0	0.0
Shellharbour (North)	0	4.0	0.0
Woolgoolga	0	4.0	0.0
South West Rocks	0	3.5	0.0
Evans Head (Airforce)	0	1.2	0.0
Port Macquarie (Town)	0	0.0	0.0
Lake Macquarie (Blacksmiths)	0	0.0	0.0

Figure S1: Total drone flights conducted for each sampling season across all beach locations. Sampling seasons are as follows: one (Sep. 2017 – May 2018), two (Sep. 2018 – May 2019), three (Sep. 2019 – May 2020) and four (Sep. 2020 – May 2021).

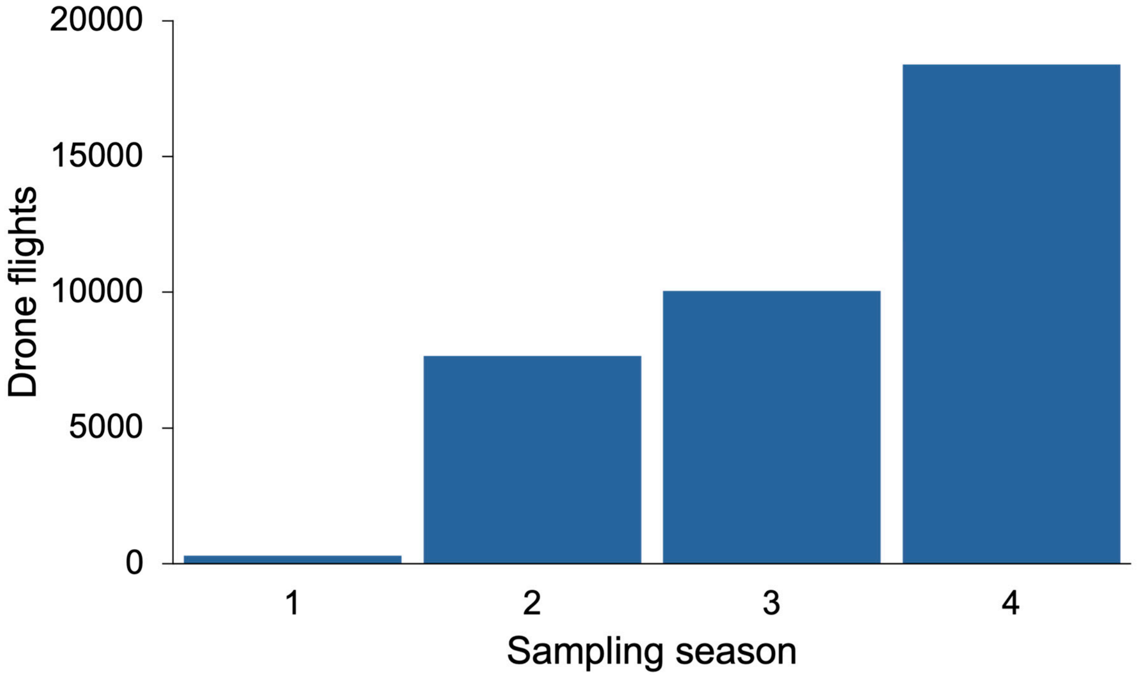

Figure S2: The total number of drone flight days with observations in relation to water clarity classifications (ranging from very poor to very good).

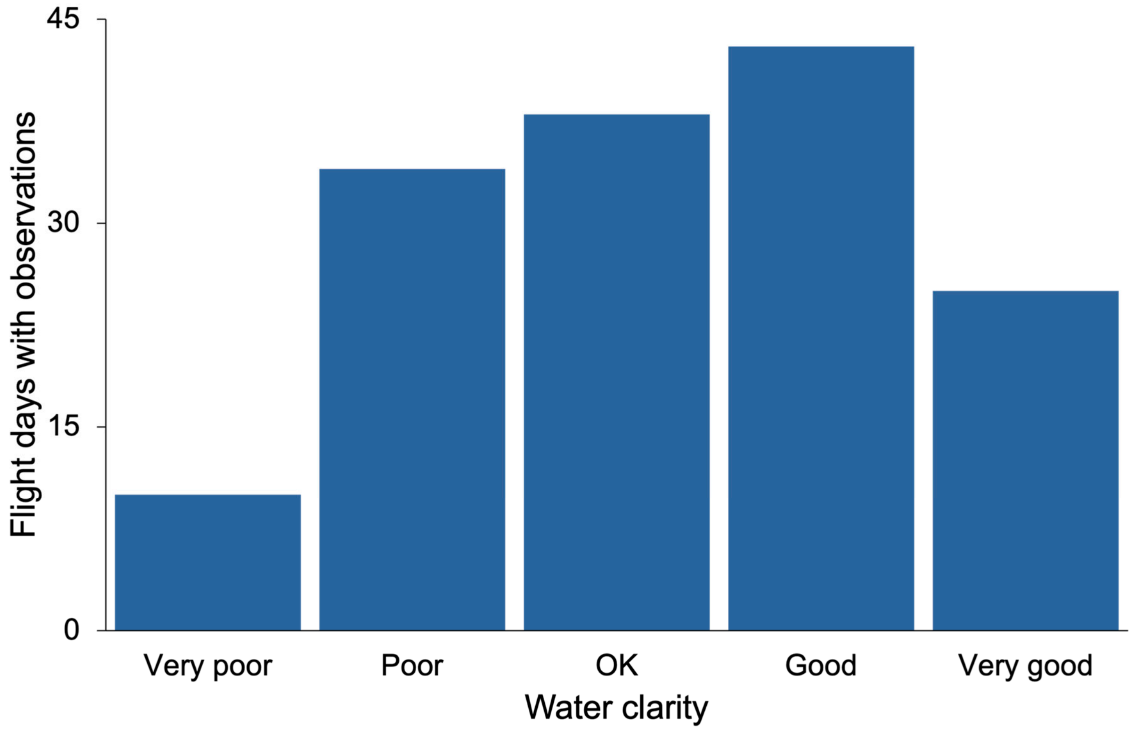

Figure S3: The total number of drone flight days with observations in relation to sea state measured using the Beaufort scale (ranging from 1 to 3).

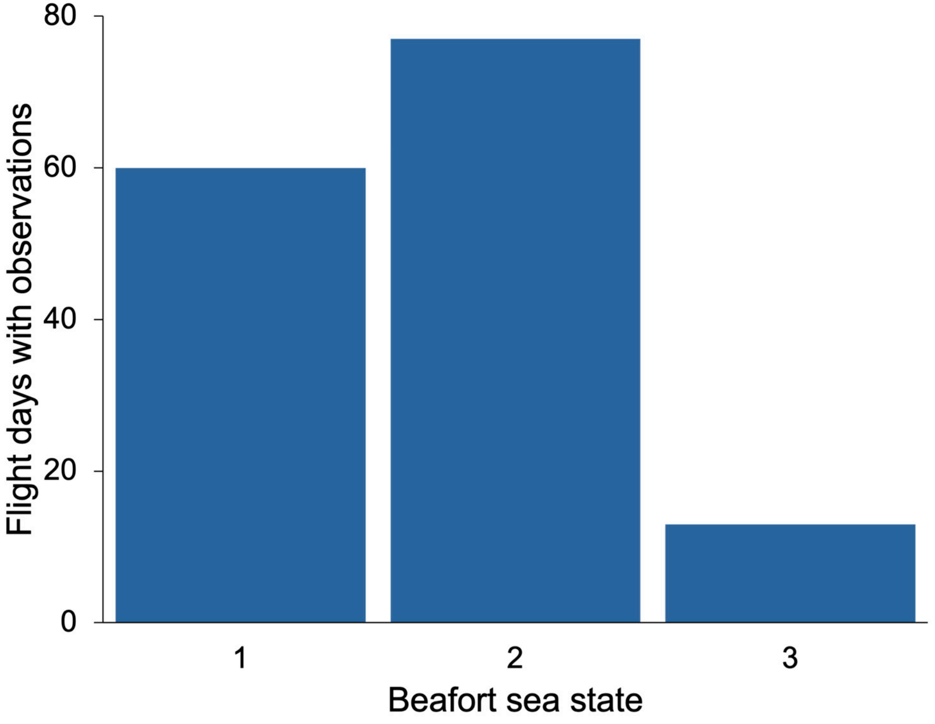

Supplement: Supplementary file 1 [file biology-11-01456-s001.zip › biology-1902029-supplementary.pdf]
